# Assessment of vitamin D nutritional status among adults in Lu'an, China, based on serum 25-hydroxyvitamin D measured by LC-MS/MS

**DOI:** 10.3389/fnut.2025.1695862

**Published:** 2026-01-16

**Authors:** Can Liu, Xianlin Zhan, Baolei Qin, Lei Sun, Rongrong Zhai

**Affiliations:** 1 Department of Clinical Laboratory, Lu'an Hospital of Anhui Medical University, Lu'an, China; 2 Department of Clinical Laboratory, Naval Medical Center, Naval Medical University, Shanghai, China

**Keywords:** age groups, central China, LC-MS/MS, seasonal variation, sex differences, vitamin D

## Abstract

**Background:**

Vitamin D deficiency is a global public health concern, yet data from central China—particularly from Lu'an, a city located within the Dabie Mountain area—remain limited.

**Objectives:**

This study aimed to evaluate vitamin D nutritional status in adults from Lu'an, China, based on serum 25-hydroxyvitamin D [25(OH)D] measured by liquid chromatography-tandem mass spectrometry (LC-MS/MS), and to assess variations by sex, age, season, and the relative contributions of 25(OH)D_2_ and 25(OH)D_3_.

**Methods:**

Serum samples from 380 adults undergoing health examinations between October 2023 and May 2025 were analyzed. Nutritional status categories and subgroup differences were compared using nonparametric methods, with stratified comparisons by sex, age, season, and body mass index.

**Results:**

The median serum 25(OH)D level was 21.60 ng/ml, with 11.05% of subjects being vitamin D deficient and 31.05% insufficient. Women had significantly lower vitamin D levels than men (*P* = 0.042), with higher deficiency and insufficiency rates (*P* < 0.001; *P* = 0.035). Although no significant differences were found in overall levels among age groups, the deficiency rate was significantly higher in young adults than in the elderly group (*P* = 0.043). Seasonal analysis showed the lowest 25(OH)D levels in winter (median 18.66 ng/ml) and the highest in summer (median 24.31 ng/ml). Overweight participants had lower 25(OH)D levels and higher insufficiency rates than those with normal weight. Lower serum 25(OH)D levels were observed in women, during winter, and among overweight individuals. Seasonal variation was pronounced, with the lowest levels in winter and the highest in summer. 25(OH)D3 was the predominant form, accounting for 98.5% of total 25(OH)D, while 25(OH)D2 contributed only 1.5% on average.

**Conclusions:**

Vitamin D insufficiency is common among adults in Lu'an, especially in women, younger individuals, during winter, and among overweight individuals. Given the predominance of vitamin D_3_, supplementation strategies should prioritize D_3_ and be tailored to demographic, seasonal, and BMI-related high-risk subgroups, providing evidence for targeted public health interventions among adults in Lu'an.

## 1 Introduction

Vitamin D is a fat-soluble vitamin that plays a critical role in regulating calcium and phosphorus metabolism (1) and maintaining bone health (2). In recent years, increasing evidence has revealed that vitamin D also participates in immune regulation (3), anti-inflammatory responses (4), antitumor activity (5), and cardiovascular protection (6). The molecular mechanisms underlying these functions involve vitamin D's role in modulating gene expression through its active form, calcitriol, which binds to the vitamin D receptor (VDR) in various tissues. This interaction influences cellular processes such as differentiation, proliferation, and apoptosis, thereby contributing to overall health and disease prevention (7). Vitamin D exists in two major forms: vitamin D_2_ (ergokalsiferol), mainly from plant-based foods and supplements, and vitamin D_3_ (kolekalsiferol), predominantly synthesized in the skin upon ultraviolet B (UVB) exposure and available from animal sources. Vitamin D_2_ and D_3_, after entering the circulation, are hydroxylated to form 25(OH)D_2_ and 25(OH)D_3_. These hydroxylated forms are widely recognized as indicators of vitamin D nutritional status and were quantified in this study using LC-MS/MS.

Factors such as environmental pollution, lifestyle changes, and poor dietary habits have led to widespread vitamin D deficiency, which has become a global public health concern (8–10). Numerous epidemiological studies have demonstrated significant regional and seasonal variations in vitamin D levels (11, 12), suggesting that supplementation strategies should be tailored to local conditions. China spans approximately five time zones from east to west and about 49 degrees of latitude from north to south. It features diverse climates and solar radiation patterns, as well as complex geographical characteristics. In addition, dietary habits and levels of socioeconomic development vary widely across regions, further influencing vitamin D nutritional status. However, data from central China remain scarce, especially from Lu'an, a city located within the Dabie Mountain area, where geographical and lifestyle characteristics may differ from other regions.

Accurate assessment of vitamin D nutritional status requires reliable and precise measurement of its circulating forms. Serum 25-hydroxyvitamin D [25(OH)D] can be measured using various methods, including immunoassays and chromatographic techniques. Among these, liquid chromatography-tandem mass spectrometry (LC-MS/MS) is considered the gold standard due to its high specificity, sensitivity, and ability to simultaneously quantify both 25(OH)D_2_ and 25(OH)D_3_ without cross-reactivity. LC-MS/MS overcomes limitations of conventional immunoassays, such as interference from other vitamin D metabolites and matrix effects, ensuring accurate determination of total vitamin D levels.

In this study, we analyzed serum 25(OH)D levels based on data collected over the past 2 years in our hospital. We aimed to evaluate vitamin D nutritional status among adults in Lu'an using LC-MS/MS. Specifically, we analyzed serum 25(OH)D concentrations over a 2-year period to describe the prevalence of vitamin D deficiency and insufficiency, examine differences by sex, age, season, and body mass index (BMI), and determine the relative contributions of 25(OH)D_2_ and 25(OH)D_3_. These findings provide region-specific evidence that may help guide targeted vitamin D supplementation and public health strategies for adults in Lu'an and similar populations in central China.

## 2 Materials and methods

### 2.1 Study population

This study was conducted in accordance with the Strengthening the Reporting of Observational Studies in Epidemiology (STROBE) guidelines (13). This retrospective observational study analyzed data from individuals aged 18 years and older who underwent serum 25(OH)D testing at the Health Management Center affiliated with Lu'an People's Hospital between October 2023 and May 2025. The center is a public facility that provides routine health examinations to adults from all four counties and three districts of Lu'an. Inpatients and disease-specific outpatient populations were excluded.

Inclusion criteria were as follows:

- Permanent residents of Lu'an aged ≥18 years;
- Serum 25(OH)D_2_ and 25(OH)D_3_ levels were both measured;
- Measurement was performed using LC-MS/MS.

Exclusion criteria included:

- Individuals with confirmed severe liver or kidney disease or those receiving medications known to affect vitamin D metabolism (e.g., phenobarbital, carbamazepine, dexamethasone);
- Incomplete basic information (i.e., missing sex, age, or test date);
- Pregnant women, patients with autoimmune diseases, or individuals with malignancies.

A total of 380 participants (214 males and 166 females) were included in the final analysis. The study protocol was approved by the Ethics Committee of Lu'an Hospital affiliated with Anhui Medical University (Approval No. 2023LLKS011), which granted a waiver of informed consent due to the retrospective and anonymized nature of the study. This study was conducted in accordance with the Declaration of Helsinki (1975, revised 2024).

### 2.2 Measurement method

Serum 25-hydroxyvitamin D [25(OH)D], including 25(OH)D_2_ and 25(OH)D_3_, was measured using liquid chromatography–tandem mass spectrometry (LC-MS/MS) following standard operating procedures. Fasting venous blood samples (≥3 ml) were collected in the morning into coagulation tubes. After standing at room temperature for 30 min to allow clotting, samples were centrifuged at 3,000 rpm for 10 min at 4 °C. Serum was analyzed within 2 h or, if delayed, stored at −20 °C and analyzed within 24 h. According to the reagent instructions, samples can be stored at 2–8 °C for up to 14 days or at −20 °C for up to 3 months, with no more than four freeze-thaw cycles.

LC-MS/MS analysis was performed using a SCIEX Triple Quad 4500MD mass spectrometer (Desci Biological Technology Co., China) with chromatographic separation on a Phenomenex column (DISIGNS column-002, Kailai Spectrum Technologies Co., China). Sample pretreatment involved protein precipitation using sulfate reagents and addition of internal standards 25(OH)D_2_-IS and 25(OH)D_3_-IS. Calibration curves were constructed using five calibrators, and two levels of quality control (QC) samples were analyzed in each batch. All calibration materials were traceable to NIST SRM 2972a certified reference material.

An in-house analytical performance verification was conducted prior to sample analysis in accordance with routine clinical laboratory practice. Precision was evaluated using quality control materials at two concentration levels. Intra-assay precision, assessed by three replicates per sample for three samples at each concentration, showed coefficients of variation (CVs) of 3.65%/0.95%/7.09% and 4.37%/1.37%/0.67% for 25(OH)D_3_, and 4.58%/3.40%/0.92% and 1.14%/1.94%/1.73% for 25(OH)D_2_ at low and high concentrations, respectively. Inter-assay precision assessed over three consecutive days demonstrated CVs of 5.05 and 3.72% for 25(OH)D_3_, and 5.26 and 2.99% for 25(OH)D_2_. Accuracy was assessed by recovery experiments using spiked serum samples, with mean recoveries of 100.35% (range: 94.4%−110.0%) for 25(OH)D_3_ and 100.19% (range: 89.8%−107.0%) for 25(OH)D_2_. These results indicate that the LC-MS/MS method demonstrated acceptable precision and accuracy for the quantification of serum 25-hydroxyvitamin D. The lower limits of quantification (LOQ) were 2.80 ng/ml for 25(OH)D_2_ and 4.50 ng/ml for 25(OH)D_3_. Detailed validation data, including precision, accuracy, and LOQ are provided in Supplementary material.

Total 25(OH)D concentration was calculated as the sum of 25(OH)D_2_ and 25(OH)D_3_. All procedures complied with internal laboratory quality assurance and manufacturer-recommended parameters to ensure analytical accuracy and reproducibility.

### 2.3 Criteria for vitamin D nutritional status

Vitamin D nutritional status was assessed based on serum total 25(OH)D concentrations, in accordance with the guidelines established by the Institute of Medicine (IOM) in the Dietary Reference Intakes for Calcium and Vitamin D (2011), which remains the most current official recommendation issued by the National Academies (14). The classification was as follows:

- Deficiency: < 12 ng/ml.
- Insufficiency: 12–20 ng/ml.
- Sufficiency: ≥20 ng/ml.

### 2.4 Statistical analysis

Statistical analysis was performed using SPSS version 25.0. The normality of continuous variables was assessed using the Shapiro–Wilk test. Serum 25(OH)D concentrations were not normally distributed, data are presented as median (P25, P75). Comparisons of serum 25(OH)D levels between two groups were conducted using the Mann–Whitney *U*-test, while comparisons among more than two groups were performed using the Kruskal–Wallis test with *post hoc* pairwise comparisons when appropriate. Vitamin D deficiency, insufficiency, and sufficiency rates were expressed as counts and percentages [*n* (%)], and group differences were analyzed using the χ^2^ or Fisher's exact test as appropriate. Given the modest sample size and unequal distribution among subgroups, multivariable regression analysis was not performed. A two-tailed *P*-value < 0.05 was considered statistically significant.

## 3 Results

### 3.1 Basic characteristics of the study population

Between October 2023 to May 2025, a total of 380 participants were included, including 214 males and 166 females, with ages ranging from 19 to 91 years. Participants' permanent residences covered the entire Lu'an area, including three urban districts (Jin'an, Yu'an, Yeji) and four counties (Huoshan, Huoqiu, Jinzhai, Shucheng).

### 3.2 Serum 25(OH)D Levels and vitamin D nutritional status by sex

The median serum 25(OH)D level of all 380 participants was 21.60 ng/ml, with 11.05% classified as deficient, 31.05% as insufficient, and 57.89% as sufficient.

Female participants had a median 25(OH)D level of 19.63 ng/ml, significantly lower than the male group's median of 22.30 ng/ml (*Z* = −2.038, *P* = 0.042). Additionally, the deficiency and insufficiency rates were significantly higher in females than males (17.47 vs. 6.07%, χ^2^ = 12.347, *P* < 0.001; 36.75 vs. 26.64%, χ^2^ = 4.464, *P* = 0.035), while the sufficiency rate was significantly lower in females compared to males (45.78 vs. 67.29%, χ^2^ = 17.738, *P* < 0.001). As shown in Table 1.

**Table 1:** Comparison of serum 25(OH)D levels and vitamin D nutritional status between sexes.

**Group**	**25(OH)D (ng/ml) *M* (P25, P75)**	**Deficiency [*n* (%)]**	**Insufficiency [*n* (%)]**	**Sufficiency [*n* (%)]**
Total (*n* = 380)	21.60 (15.65, 28.53)	42 (11.05%)	118 (31.05%)	220 (57.89%)
Male (*n* = 214)	22.30 (18.18, 27.08)	13 (6.07%)	57 (26.64%)	144 (67.29%)
Female (*n* = 166)	19.63 (13.47, 30.29)	29 (17.47%)	61 (36.75%)	76 (45.78%)
*Z*/χ^2^	−2.038	12.347	4.464	17.738
*P*	0.042	< 0.001	0.035	< 0.001

Z values are from Mann–Whitney U-test; χ^2^ values are from χ^2^ test. P < 0.05 was considered statistically significant.

### 3.3 Serum 25(OH)D levels and vitamin D nutritional status among different age groups

To explore the effect of age on 25(OH)D levels, participants were divided into three groups: young adults (18–39 years), middle-aged adults (40–59 years), and older adults (≥60 years). The median serum 25(OH)D levels were 20.08, 21.71, and 23.47 ng/ml for the young, middle-aged, and older groups, respectively. No statistically significant differences were found between any two groups (*P* > 0.05).

The deficiency rate in the young group was significantly higher than that in the older group (16.54 vs. 7.45%, χ^2^ = 4.088, *P* = 0.043). The sufficiency rate in the middle-aged group was significantly higher than in the young group (63.40 vs. 50.38%, χ^2^ = 4.933, *P* = 0.026). The insufficiency rates for the young, middle-aged, and older groups were 33.08%, 28.10%, and 32.98%, respectively, with no significant differences among groups (*P* > 0.05). As shown in Table 2.

**Table 2:** Comparison of serum 25-hydroxyvitamin D levels and vitamin D nutritional status among different age groups.

**Group**	**25(OH)D (ng/ml) M (P25, P75)**	**Deficiency [*n* (%)]**	**Insufficiency [*n* (%)]**	**Sufficiency [*n* (%)]**
Young (*n* = 133)	20.08 (13.53, 29.06)	22 (16.54%)^*^	44 (33.08%)	67 (50.38%)^#^
Middle-aged (*n* = 153)	21.71 (17.69, 26.82)	13 (8.50%)	43 (28.10%)	97 (63.40%)
Older (*n* = 94)	23.47 (15.75, 30.18)	7 (7.45%)	31 (32.98%)	56 (59.57%)

^*^Compared to the older group, P < 0.05.

^#^Compared to the middle-aged group, P < 0.05.

### 3.4 Serum 25-hydroxyvitamin D levels and vitamin D nutritional status among different seasons

We further analyzed 25(OH)D levels across different seasons. The seasonal classification in China is defined as: spring (March–May), summer (June–August), autumn (September–November), and winter (December–February of the following year). Results showed that serum 25(OH)D levels were lowest in winter (median 18.66 ng/ml), followed by spring and autumn (medians of 20.55 and 22.66 ng/ml, respectively), and highest in summer (median 24.31 ng/ml). Compared with winter, 25(OH)D levels were significantly higher in summer (*Z* = −3.543, *P* < 0.001) and autumn (*Z* = −3.402, *P* = 0.001). In addition, summer levels were also significantly higher than those in spring (*Z* = −2.122, *P* = 0.034).

The deficiency rate was highest in winter (23.46%), followed by spring (12.63%), and lowest in autumn and summer (5.19 and 5.80%, respectively), with autumn significantly lower than spring (χ^2^ = 4.080, *P* = 0.043) and winter (χ^2^ = 15.962, *P* < 0.001), and summer significantly lower than winter (χ^2^ = 8.951, *P* = 0.003). The insufficiency rate was lowest in summer (26.09%), whereas spring, autumn, and winter showed rates of 33.68%, 31.85%, and 30.86%, respectively, with no significant differences among groups (*P* > 0.05). The sufficiency rate was lowest in winter (45.68%), followed by spring (53.68%) and autumn (62.96%), and highest in summer (68.12%), with summer and autumn showing significantly higher sufficiency than winter (χ^2^ = 7.613, *P* = 0.006; χ^2^ = 6.153, *P* = 0.013). As shown in Table 3.

**Table 3:** Comparison of serum 25-hydroxyvitamin D levels and vitamin D nutritional status among different seasons.

**Group**	**25(OH)D (ng/ml) *M* (P25, P75)**	**Deficiency [*n* (%)]**	**Insufficiency [*n* (%)]**	**Sufficiency [*n* (%)]**
Spring (*n* = 95)	20.55 (13.72, 27.38)	12 (12.63%)	32 (33.68%)	51 (53.68%)
Summer (*n* = 69)	24.31 (19.07, 29.51)^a, b^	4 (5.80%)^c^	18 (26.09%)	47 (68.12%)^c^
Autumn (*n* = 135)	22.66 (16.99, 29.57)^c^	7 (5.19%)^a, b^	43 (31.85%)	85 (62.96%)^c^
Winter (*n* = 81)	18.66 (12.03, 25.84)	19 (23.46%)	25(30.86%)	37 (45.68%)

^a^Compared with spring, P < 0.05.

^b^Compared with winter, P < 0.001.

^c^Compared with winter, P < 0.05.

### 3.5 Association of serum 25(OH)D levels with BMI categories

To assess the potential influence of adiposity on vitamin D status, participants were categorized into BMI groups according to both Chinese and WHO criteria.

Using the Chinese BMI criteria (15), participants were grouped into underweight (BMI < 18.5 kg/m^2^), normal weight (18.5 ≤ BMI < 24 kg/m^2^), overweight (24 ≤ BMI < 28 kg/m^2^), and obese (BMI ≥ 28 kg/m^2^) categories. The median serum 25(OH)D level in the overweight group was significantly lower than that in the normal-weight group (18.53 vs. 22.68 ng/ml, *P* < 0.05), while the underweight and obese groups did not show statistically significant differences. The insufficiency rate was higher in the overweight group compared with the normal-weight group (44.56 vs. 27.57%, *P* < 0.05). As shown in Table 4.1.

**Table 4.1:** Serum 25(OH)D levels and vitamin D status by Chinese BMI criteria.

**Group**	**25(OH)D (ng/ml) *M* (P25, P75)**	**Deficiency [*n* (%)]**	**Insufficiency [*n* (%)]**	**Sufficiency [*n* (%)]**
Underweight (*n* = 9)	22.87 (19.68, 27.84)	1 (11.11%)	1 (11.11%)	7 (77.78%)
Normal-weight (*n* = 272)	22.68 (16.19, 29.28)	25(9.19%)	75 (27.57%)	172 (63.24%)
Overweight (*n* = 92)	18.53 (14.36 23.90)^#^	14 (15.22%)	41 (44.56%)^#^	37 (40.22%)^#, *^
Obese (*n* = 7)	25.34 (11.69, 39.99)	2 (28.57%)	1 (14.29%)	4 (57.14%)

^#^Compared with normal group, P < 0.05.

^*^Compared with underweight group, P < 0.05.

Fisher's exact test was used for comparisons involving underweight and obesity groups due to small sample sizes.

Using the WHO BMI criteria (16), participants were grouped into underweight (BMI < 18.5 kg/m^2^), normal weight (18.5 ≤ BMI < 25 kg/m^2^), overweight (25 ≤ BMI < 30 kg/m^2^), and obese (BMI ≥ 30 kg/m^2^) categories. Similar trends were observed. The overweight group demonstrated significantly lower 25(OH)D levels compared to the normal-weight group (18.53 vs. 22.46 ng/ml, *P* < 0.05). No participants met the criteria for obese (BMI ≥ 30 kg/m^2^). As shown in Table 4.2.

**Table 4.2:** Serum 25(OH)D levels and vitamin D status by WHO BMI criteria.

**Group**	**25(OH)D (ng/ml) M (P25, P75)**	**Deficiency [*n* (%)]**	**Insufficiency [*n* (%)]**	**Sufficiency [*n* (%)]**
Underweight (*n* = 9)	22.87 (19.68, 27.84)	1 (11.11%)	1 (11.11%)	7 (77.78%)
Normal-weight (*n* = 287)	22.46 (16.23, 29.22)	27 (9.41%)	81 (28.22%)	179 (62.37%)
Overweight (*n* = 84)	18.53 (13.58 25.24)^#^	14 (16.67%)	36 (42.86%)^#^	34 (40.48%)^#, *^
Obese (*n* = 0)	–	–	–	–

^#^Compared with normal group, P < 0.05.

^*^Compared with underweight group, P < 0.05.

Obese group included no participants; Fisher's exact test used for comparisons involving underweight group.

### 3.6 Relative contribution of 25(OH)D_2_ and 25(OH)D_3_ to total 25(OH)D

Since 25(OH)D is the sum of 25(OH)D2 and 25(OH)D3, to quantify their relative contributions, we calculated the percentage of vitamin D_2_ and D_3_ in the total vitamin D. The results showed that the average contribution of 25(OH)D3 was approximately 98.5% (ranging from about 95.6 to 99.9%), while the contribution of 25(OH)D2 was generally small, averaging about 1.5% (ranging from approximately 0.1 to 4.4%). Among the 380 participants, 60 individuals had a 25(OH)D2 contribution greater than 10%, and 12 individuals had a 25(OH)D2 contribution exceeding 50%.

## 4 Discussion

This study utilized liquid chromatography-tandem mass spectrometry (LC-MS/MS) technology to measure serum 25(OH)D levels in 380 adults from Lu'an for the first time. Assay standardization is crucial for comparability across studies. This method is recognized as the gold standard for measuring vitamin D levels due to its high accuracy and reliability. We analyzed vitamin D nutritional status and examined the effects of sex, age, season, and body mass index (BMI). In addition, we assessed the relative contributions of 25(OH)D_2_ and 25(OH)D_3_. The median serum 25(OH)D level in the study population was 21.60 ng/ml, with 11.05% classified as vitamin D deficient (< 12 ng/ml) and 31.05% as insufficient (12–20 ng/ml). Thus, 42.10% of individuals had low vitamin D nutritional status (< 20 ng/ml), and only 57.89% maintained sufficient levels, indicating that the vitamin D nutritional status of adults in Lu'an remains suboptimal. This observation is consistent with a large-scale systematic review and meta-analysis of Chinese populations, which reported an overall mean 25(OH)D concentration of 44.3 nmol/L (~17.7 ng/ml) in adults and a pooled prevalence of vitamin D deficiency (< 30 nmol/L) of 20.7% and insufficiency (< 50 nmol/L) of 63.2% in Mainland China (17). We focused on the influence of variables such as sex, season, age, and BMI on vitamin D nutritional status, as well as the contribution ratio of D_2_ and D_3_.

Regarding sex, females had significantly lower median 25(OH)D levels (19.63 ng/ml) than males (22.30 ng/ml), with higher deficiency and insufficiency rates. These findings are appropriate and can reinforce a recent study of 1,528,685 samples from 30 provinces of China measured by LC-MS/MS, which reported higher median 25(OH)D levels in males (25.5 ng/ml) than in females (20.8 ng/ml) (18). This may be related to women spending less time outdoors, having shorter sun exposure durations, wearing more covering clothing during outdoor activities, and paying more attention to UV protection, all of which inhibit cutaneous vitamin D synthesis to some extent. Additionally, women often emphasize body shape and may have unbalanced diets, including dieting, picky eating, or even strict vegetarianism, which limits fat intake and thus reduces absorption of fat-soluble vitamin D. Similar findings have been reported in epidemiological studies from Beijing (11), Shanghai (12), and other regions internationally, indicating a consistent sex-related pattern in vitamin D metabolism and exposure behavior. Therefore, women should be particularly attentive to vitamin D supplementation and intake in daily life.

It is generally recognized that aging reduces cutaneous vitamin D synthesis due to lower 7-dehydrocholesterol levels and diminished photosynthetic capacity, typically resulting in lower serum 25(OH)D concentrations in the elderly. However, this age-related decline was not evident in our study population. In univariate analyses, older adults (≥60 years) showed slightly higher median 25(OH)D levels and a lower deficiency rate compared with younger adults. These findings suggest that factors other than age alone may contribute to the observed pattern. Older adults in Lu'an generally maintain stable daily routines and engage in regular light outdoor activity, which may increase sunlight exposure. The area is also known for favorable air quality, potentially enhancing effective UVB exposure. Traditional dietary patterns, including regular intake of dairy products and locally consumed foods rich in antioxidants (19, 20), may also support overall nutritional status, although their direct effects on vitamin D concentrations remain uncertain. Future studies incorporating detailed lifestyle, dietary, and environmental assessments are needed to clarify the determinants of vitamin D levels across age groups in this region.

In addition to sex, age, and season, BMI was identified as an important factor influencing vitamin D status. Participants with normal body weight showed higher serum 25(OH)D levels and lower prevalence of insufficiency compared with overweight individuals. These results are consistent with prior evidence that excess adiposity sequesters vitamin D in adipose tissue, reducing its bioavailability (21). Although no participants reached the WHO-defined obesity threshold (BMI ≥30 kg/m^2^), the data suggest that even moderate increases in BMI are associated with lower vitamin D levels, highlighting overweight adults as a high-risk group for vitamin D deficiency. Public health interventions should therefore consider BMI when planning screening and supplementation strategies.

Vitamin D synthesis depends on UVB exposure, exhibiting marked seasonal variation, which our study confirmed. 25(OH)D levels peaked in summer (median 24.31 ng/ml) and were lowest in winter (median 18.66 ng/ml), showing a typical seasonal fluctuation consistent with sun-dependent synthesis. Longer summer daylight, stronger UV radiation, and more outdoor activity promote cutaneous vitamin D production, while weaker winter sunlight, heavy clothing, and reduced outdoor activity limit synthesis and reduce serum levels. Interestingly, though autumn has shorter daylight than summer, vitamin D deficiency rates were slightly lower in autumn (5.19%) than summer (5.80%). Some studies indicate that extremely high temperatures may reduce vitamin D levels by discouraging outdoor activities (22), suggesting that sunlight duration alone is insufficient to guide supplementation. Moreover, excessive UV exposure increases skin cancer risk, so excessive sunbathing is not recommended. Clinically, attention should be paid to seasonal influences on vitamin D levels, especially during winter, to enhance screening and intervention for high-risk groups and reduce deficiency prevalence. The observed variations in serum 25(OH)D levels according to sex, age, and season were consistent with international reports, supporting the reliability of our results and suggesting that similar influencing factors operate in the Dabie Mountains region of China. Such seasonal variation has also been documented in southern China, where high-accuracy LC-MS/MS measurement revealed considerable fluctuation of 25(OH)D status across seasons, underscoring the influence of geographic, latitudinal and exposure factors (23).

Analysis of 25(OH)D_2_ and 25(OH)D_3_ contributions revealed that D_3_ accounted for 98.5% of total 25(OH)D, whereas D_2_ contributed only 1.5%, with few individuals exceeding 10% D_2_. This indicates that vitamin D in this population mainly originates from vitamin D_3_, reflecting the dominant role of animal food intake and UV exposure in vitamin D generation, with limited contribution from plant sources or supplements containing vitamin D_2_. Multiple studies (24, 25) have also shown that vitamin D_3_ is more effective than D_2_ in raising serum vitamin D levels. For vegetarians, increasing outdoor activities to enhance cutaneous D_3_ synthesis and consuming mushrooms rich in D_2_ can raise serum vitamin D. Interestingly, fresh mushrooms have low D_2_, but UV exposure significantly increases their D_2_ content (26), and consuming UV-treated mushrooms effectively raises serum D_2_ in consumers (27). Therefore, we recommend prioritizing vitamin D_3_-fortified foods or sun-dried mushrooms in nutritional interventions to optimize supplementation.

This study provides valuable data on serum 25(OH)D levels in Lu'an, but has several limitations. Participants were recruited from a hospital-based health examination population, which may not fully represent all adults in Lu'an or the broader Dabie Mountain region, although the hospital serves multiple counties. The total sample size (*n* = 380) was modest, and some subgroup sizes were unbalanced, which may affect the precision of comparisons; non-parametric tests were applied to mitigate these effects. The cross-sectional design precludes causal inference, and longitudinal studies are needed to clarify temporal relationships. Additionally, detailed lifestyle and environmental factors—including diet, supplementation, physical activity, sun exposure, skin pigmentation, and socioeconomic status—were not assessed, all of which can influence 25(OH)D levels. The modest sample size and unbalanced subgroup distribution limited the feasibility of multivariable modeling; therefore, analyses were restricted to descriptive and stratified comparisons. Future studies with larger, balanced, and population-based cohorts, incorporating these variables, are warranted to validate and extend the current findings and guide targeted vitamin D interventions.

Our findings indicate that vitamin D insufficiency is common among adults in Lu'an, particularly in women, younger individuals, overweight participants, and during the winter months. Given that 25(OH)D_3_ accounts for the vast majority of circulating vitamin D, supplementation strategies should focus on vitamin D_3_. We recommend targeted interventions for these high-risk subgroups, including daily supplementation with 800–1,000 IU of vitamin D_3_ during the winter season (October–March). Routine health check-ups for these populations could incorporate screening for vitamin D deficiency to enable timely intervention and follow-up. Furthermore, local health authorities could promote awareness of vitamin D insufficiency, integrate supplementation guidance into community health programs, and consider establishing a serum 25(OH)D monitoring system to evaluate intervention effectiveness. These recommendations provide concrete, evidence-based, and actionable steps for improving vitamin D status and supporting public health in Lu'an.

In summary, this study describes the vitamin D status of adults in Lu'an and the influence of sex, age, season, and BMI. Vitamin D insufficiency is particularly common in women, overweight individuals, younger adults, and during winter. Targeted interventions, including seasonal supplementation with vitamin D_3_ and routine screening for high-risk groups, are recommended. Public health campaigns and community programs could further raise awareness and support improvement of vitamin D status in this population.

## Data Availability

The original contributions presented in the study are included in the article/Supplementary material, further inquiries can be directed to the corresponding author.
